# The Alcohol Treatment of Burns

**Published:** 1913-04-19

**Authors:** 


### The Alcohol Treatment of Burns.

Steadily the treatment of burns has emerged from the rough-and-ready stage of carron oil and similar septic relics of the pre-Listerian epoch; but finality has not yet been reached by a long way, and each of the four or five commonly used methods of treatment has drawbacks and disadvantages. At one time, about nine or ten years ago, the very heroic treatment was advocated of scrubbing the whole burnt surface (under anæsthesia) with a strong antiseptic and a nail-brush. This had the sanction of some of the highest surgical authorities; but experience soon decided that the plan is too drastic, and that it does not even assure the main object which it was designed for, an aseptic condition of the burn. Of the dressings introduced of late years, picric acid has still many supporters, though several cases of picric acid poisoning have been reported. A bath of warm normal salt solution for the whole affected surface, kept constantly at body heat, alternating with sterile dressings soaked in the same fluid, produces wonderful results in very severe cases; but is, of course, extremely difficult and troublesome to carry out even in hospital.

In a recent paper read before the Pediatric Society in Melbourne, Dr. Milligan reports with enthusiasm upon the "alcohol treatment." This method has certain drawbacks, but the countervailing advantages more than offset them. The routine as used in the Melbourne Children's Hospital is as follows:

If the patient is too much "shocked" to bear an anæsthetic, a saturated watery solution of picric acid on lint is applied, and covered with protective. The next day more picric acid solution is poured on, without changing the dressing. In other words, the ordinary picric acid treatment is adopted. But as soon as the shock has sufficiently passed off to make administration of an anæsthetic safe, chloroform is given: ether would seem to be an obvious improvement, but Dr. Milligan expresses a preference for the less safe drug. The burns are then cleaned up with 70 per cent. alcohol. Sterile gauze is soaked in this and all blisters and dead tissue are vigorously rubbed away. After this a dressing of sterilised gauze soaked in 70 per cent. alcohol is applied, and covered with dry gauze, wool, and a bandage.

This process is repeated daily under an anæsthetic. The dressing is often found stuck to the surface, and when pulled off it brings away bits of dead tissue with it. The remainder of the necrosed tissue is removed by the process of rubbing with gauze. These daily dressings are continued for about eight days, and burns of the second or third degree are then as a rule so clean and healthy that simple boroglyceride dressings can be used without pain to the patient, and with every prospect of rapid healing. If the burns are deeper, the alcohol treatment must be continued for a longer period. Sloughs are not allowed to separate, but are removed by cutting with a scalpel. Owing to their dry and shrivelled state after the alcohol treatment, they are not septic; and therefore there is no danger in this radical way of removing them.

First and foremost, the need for a daily anæsthetic necessitated owing to the painfulness of the method is a drawback that is undeniable. In practice, Dr. Milligan says, it does not harm, and does not even cause vomiting; on general principles the use of chloroform, especially for children, would seem less desirable than that of ether. It is stated that seven or eight times is the average number of administrations required for each case.

The next objection is that in some cases of extensive burnt surfaces, absorption of alcohol has been sufficiently great to set up deep alcoholic coma. This can be prevented, and the treatment made not any less effective, by placing a layer of boroglyceride gauze next the burnt surface, and a single layer of gauze wrung out of alcohol over this.

The third disadvantage is the matter of expense. 70 per cent. alcohol costs about a sovereign a gallon.

